# The emerging role of microRNA-4487/6845-3p in Alzheimer’s disease pathologies is induced by Aβ25–35 triggered in SH-SY5Y cell

**DOI:** 10.1186/s12918-018-0633-3

**Published:** 2018-12-14

**Authors:** Ling Hu, Rong Zhang, Qiong Yuan, Yinping Gao, Mary Q. Yang, Chunxiang Zhang, Jiankun Huang, Yufei Sun, William Yang, Jack Y. Yang, Zhen-li Min, Jing Cheng, Youping Deng, Xiamin Hu

**Affiliations:** 1 Department of Anesthesiology, Tianyou Hospital, Wuhan University of Science and Technology, Wuhan, 430064 China; 2 Department of Pharmacy, College of Medicine, Wuhan University of Science and Technology, Wuhan, 430065 Hubei Province China; 3 Department of Pharmacy, Shanghai University of Medicine & Health Sciences, Shanghai, 201318 China; 4 MidSouth Bioinformatics Center, Department of Information Science, George Washington Donaghey College of Engineering and Information Technology and Joint Bioinformatics Graduate Program, University of Arkansas at Little Rock and University of Arkansas for Medical Sciences, Little Rock, AR 72204 USA; 5 Department of Biomedical Engineering, School of Medicine and School of Engineering, The University of Alabama, Birmingham, 35201 USA; 6 Bioinformatics Core, Department of Complementary & Integrative Medicine, University of Hawaii John A. Burns School of Medicine, Honolulu, HI 96813 USA

**Keywords:** Alzheimer, miR-4487, miR-6845-3p, Apoptosis, Axon

## Abstract

**Background:**

Accumulation of amyloid β-peptide (Aβ) is implicated in the pathogenesis and development of Alzheimer’s disease (AD). Neuron-enriched miRNA was aberrantly regulated and may be associated with the pathogenesis of AD. However, regarding whether miRNA is involved in the accumulation of Aβ in AD, the underlying molecule mechanism remains unclear. Therefore, we conduct a systematic identification of the promising role of miRNAs in Aβ deposition, and shed light on the molecular mechanism of target miRNAs underlying SH-SY5Y cells treated with Aβ-induced cytotoxicity.

**Results:**

Statistical analyses of microarray data revealed that 155 significantly upregulated and 50 significantly downregulated miRNAs were found on the basis of log2 | Fold Change | ≥ 0.585 and *P <* 0.05 filter condition through 2588 kinds of mature miRNA probe examined. PCR results show that the expression change trend of the selected six miRNAs (miR-6845-3p, miR-4487, miR-4534, miR-3622-3p, miR-1233-3p, miR-6760-5p) was consistent with the results of the gene chip. Notably, Aβ_25–35_ downregulated hsa-miR-4487 and upregulated hsa-miR-6845-3p in SH-SY5Y cell lines associated with Aβ-mediated pathophysiology. Increase of hsa-miR-4487 could inhibit cells apoptosis, and diminution of hsa-miR-6845-3p could attenuate axon damage mediated by Aβ_25–35_ in SH-SY5Y.

**Conclusions:**

Together, these findings suggest that dysregulation of hsa-miR-4487 and hsa-miR-6845-3p contributed to the pathogenesis of AD associated with Aβ25–35 mediated by triggering cell apoptosis and synaptic dysfunction. It might be beneficial to understand the pathogenesis and development of clinical diagnosis and treatment of AD. Further, our well-designed validation studies will test the miRNAs signature as a prognostication tool associated with clinical outcomes in AD.

## Background

Alzheimer’s disease(AD), with main clinical features of progressive disorder in cognitive and behavioral functions, is the most common degenerative neurodegenerative disease. It remains at a high mortality rate worldwide, with patients suffering from this disease progressing to dementia caused by advanced neuronal dysfunction, and trends predict the disease rate to increase eighty-five times by 2050 [1]. AD is viewed as a late stage of the disease because available interventions are most likely too late to ameliorate the condition [2]. Thus, it is necessary to explore detection biomarkers as soon as possible, and to understand the development mechanism of AD.

Recently, microRNAs (miRNAs) as a class of small non-coding RNAs have expanded the horizon for neurological disease prediction and provide an efficient approach to the disease treatment of AD. Emerging evidence has demonstrated that over 70% of significant miRNAs are detected in central nervous system diseases including AD [3], stroke, and Parkinson’s, contributing to regulation of a more diverse set of cellular mechanisms such as development, synaptic plasticity secretion of neurotransmitter, and neuron survival [4–6]. Especially, functional studies have further underlined that point that the important roles of these miRNAs might be closely related to the changes of Aβ formation [7].

Interestingly, the predominant theory for AD is the “amyloid-β hypothesis”, which states that abnormally histopathological features of Aβ binds to, accumulates, and aggregates in synapses resulting in the production of a series of aggregates that are neurotoxic [8]. Studies have shown that cognitive disorder in AD derives from impaired synaptic plasticity by aberrant amyloid beta peptide [9]. Although these studies screened out several miRNAs associated with AD, the concreted pathogenesis studies on AD such as for synaptic dysfunctions remain poorly known.

Overall, two questions are resolved in this project: whether miRNAs were involved in the accumulation of Aβ in AD, and how to work the underlying molecule mechanism on apoptosis and axon damage of promising miRNAs mediated by Aβ_25–35_ in SH-SY5Y.

## Materials and methods

### Cell culture and treatment

The SH-SY5Y human neuroblastoma cell line was obtained from American type culture collection (ATCC) and maintained in high glucose modified Eagle’s medium (DMEM) supplemented with 15% fetal bovine serum and penicillin/streptomycin (100 μg/ml) at 37 °C with 5% CO_2_. The medium was refreshed once a day during cell growth. When cells reached 80~ 90% confluence, cell suspension was sub-cultured on flask according to 1:3 proportion. Cells were plated in a 96-well plate at a concentration of 1 × 10^4^ cells in per well and cultured for 24 h. Then, the medium with the concentration of Aβ25–35 (5 μM/L, 10 μM/L, 20 μM/L, 40 μM/L) was added into the culture plate for 48 h. And the physiological saline acted as the negative control.

### Cell viability assay

After the above SH-SY5Y cell in 96-well plate with different concentration of Aβ25–35 was cultured for 48 h, 20 μL MTT (5 mg/mL) was added to each well and incubated for another 4 h followed by 150 μL DMSO. After treatment, the plates were shocked for 10 min until the crystal was dissolved. The cell viability was calculated by the formulas ([normal OD-control OD/experimental OD-control OD] *100%).

### miRNA microarray and quantitative real-time PCR for miRNA verification

Total RNAs were extracted from the cells of Aβ25–35 group and control group using Trizol reagent (Invitrogen, Carlsbad, CA, USA) according to the manufacturer’s instructions. Quality of RNA was determined using an Agilent 2100 Bioanalyzer (Agilent Technologies). The integrity number and quality of RNAs are related positively. Only an RNA integrity number ≥ 5 were used for further analyses. The coefficient variance(CV) of repeated probe were calculated. The next microarray analysis was processed by invariant set normalization method. The miRNAs which the difference in its expression was more than 2.0-fold were considered to be significant gene.

Each qRT-PCR assay was performed in triplicate assay in accordance with the specifications of miRNA qRT-PCR SYBR® Kit (Takara). Synthetic cDNA was amplified specifically and quantitatively using a miRNA-specific primer and SYBR advantage qPCR chemistry. The primers of quantitative PCR were synthesized by Ribo Biotech Co (Shanghai). The reaction conditions were as follows: 95 °C for 2 min followed by 40 cycles of 95 °C for 10 s, 60 °C for 15 s and 70 °C for 10s. At the end of the PCR cycling, 65 °C–95 °C melt curve analysis was performed to validate the specific generation of the expected PCR product. U6 small nuclear non-coding RNA served as an internal control.

### miRNA function analysis, Cell transfection and quantitative real-time PCR for miRNA verification

Targetscan, miRDB, miRWalk and miranda were used for prediction target gene of Each miRNA. The predicted data were combined by these algorithms, and the intersection elements were recognized as candidate target genes. The Gene Ontology (GO) database and the Kyoto Encyclopedia Genes and Genomes (KEGG) database were devoted to functional analysis of the selected target genes, in which GO terms were significantly enriched in the predicted target gene of the miRNA compared with the corresponding gene background and certain reference biological functions, and KEGG pathways were assigned to decipher the pathway and associated biological functions.

Cells were seeded in six-well across the day until cells reached 60–70% confluence. The transfection agent X-treme GENE HP DNA (Roche, Co)was deployed to the mixture of mimic/inhibitor and DMEM free of serum. Next, transfection was performed following the manufacturer’s protocol with transfection agent X-treme GENE HP DNA in room temperature. After 48 h, the cells were treated with the TRIzol reagent and stored at − 80 °C until RNA extraction according to previous method and following experiments.

### Flow cytometric detection of apoptosis

The cells from each group were collected following trypsin free of EDTA treatment gently and were centrifuged at 2000 rpm for 5 min and washed twice with PBS. The cells were re-suspended with the binding buffer and 100 μl (a final concentration of 10^6^ cells/ml) suspended cells were added to negative control tube one, negative control two and positive control tube one, positive control tube two. Then, when all of tubes were mixed to 5 μl Annexin V-FITC staining solution, the cells were subsequently incubated at 2–8 °C in the dark for 5 min and the apoptotic rates were detected using a flow cytometer (BD Biosciences, Franklin Lakes, USA). The data were analyzed using CellQuest Pro 3.3 software (BD Biosciences).

### Immunofluorescence imaging

Mir-6845-3p inhibitor and inhibitor control were transfected into SH-SY5Y cells and then treated with Aβ25–35 for 48 h. The cells were washed with PBS twice and fixed with 4% paraformaldehyde for 10 min. After they were permeabilized with 0.5% Triton-X100 for 15 min, and blocked by the goat serum. Then, Neurofilament medium protein Monoclonal antibodies, Goat Anti-rabbit IgG/FITC (Abcam, Co) of 1% BSA and DAPI gradually were put according to manufacturer’s instruction. The cells were mounted in the 50% glycerin shield, and the fluorescence images were taken using a confocal laser scanning microscope (Olympus, Japan).

### Statistical analysis

Data are presented as the mean ± standard deviation and were compared with Student’s t-tests between two groups or one-way analysis of variance (ANOVA) between three or more groups using SPSS software (version 12). *P* < 0.05 was considered to be a statistically significant difference).

## Results

### Effects of Aβ25–35 on the SH-SY5Y cell viability

**Fig. 1:**
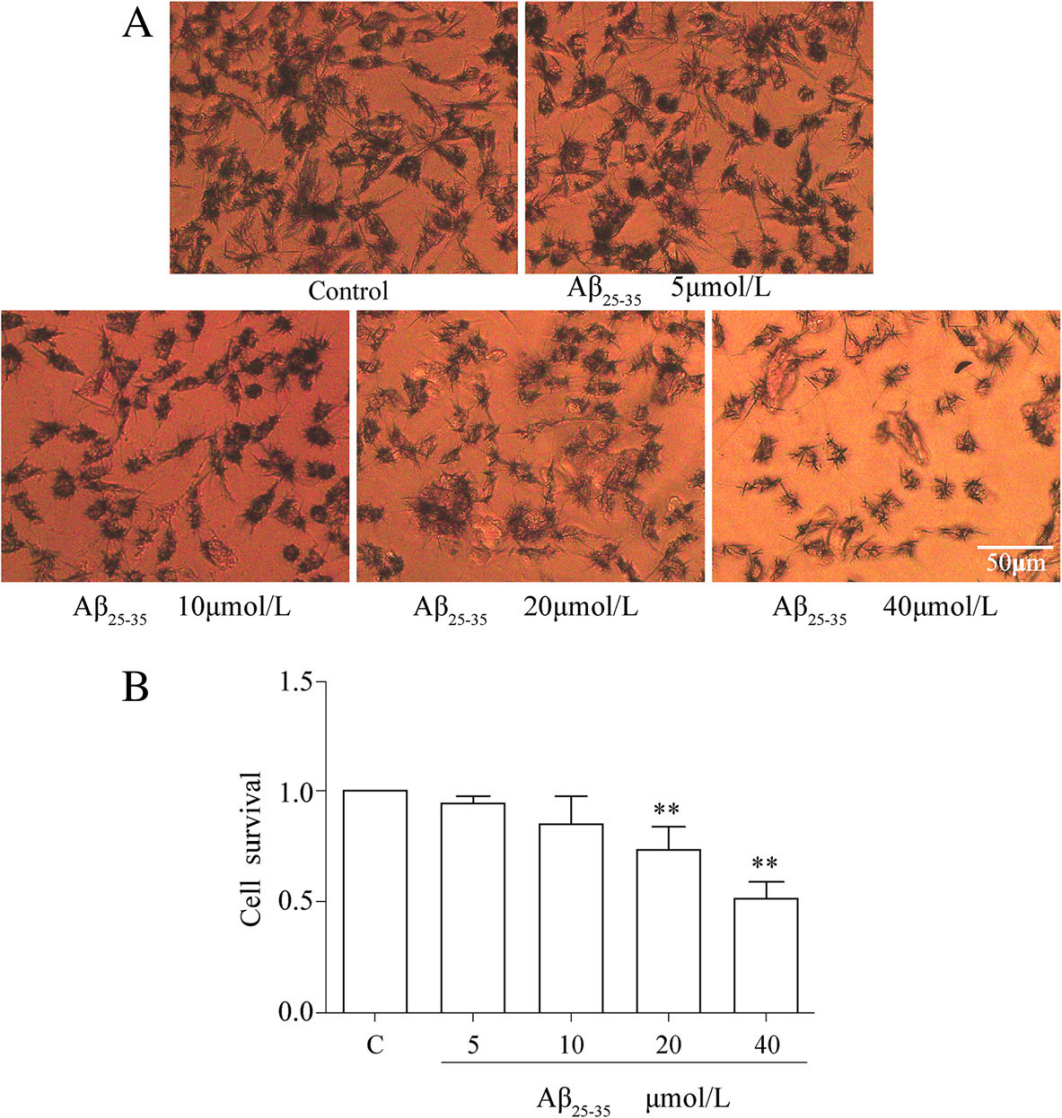
Effect of Aβ25–35 on cell viability in SH-SY5Y cells in a dose-dependent manner detected by MTT assay. **a** The representative pictures of formazan crystals treated by Aβ25–35 at different concentration taken by optical microscope (× 10). **b** The summary data of MTT. **p < 0.01 vs. control; *n* = 3

To investigate the effect of Aβ25–35 on SH-SY5Y cell viability, the MTT assay was conducted to observe the cell survival as correlated with different concentrations of Aβ25–35 treatment (5 μM/L, 10 μM/L, 20 μM/L, 40 μM/L) for 48 h. The MTT results indicated that the cell viability treated with low dose Aβ25–35 (5 μM/L and 10 μM/L) had no obvious influence. While high dose Aβ25–35 (20 μM/L and 40 μM/L) had significantly dose-dependent relationship on SH-SY5Y cell viability (Fig. 1), the cell viability with Aβ25–35 treatment was significantly decreased to 51.6% ± 7% compared with the negative control. As it is shown, cells presented the characteristic tips of formazan crystals. (Fig. 1; *P* < 0.05) and Aβ25–35 treatment (40 μM/L) group has excellent photo transmittance. 40 μM/L concentrations Aβ25–35 were selected for further studies based on the maximum valid dose and for cell viability [10].

### miRNA expression profile of SH-SY5Y cells treated with the Aβ25–35

**Fig. 2:**
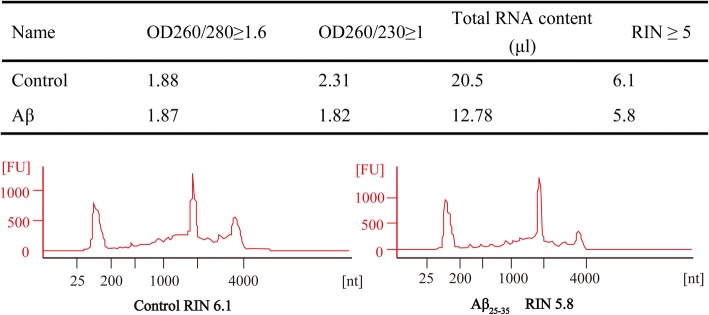
RIN value detected by Agilent 6000 Nano RNA assay

**Fig. 3:**
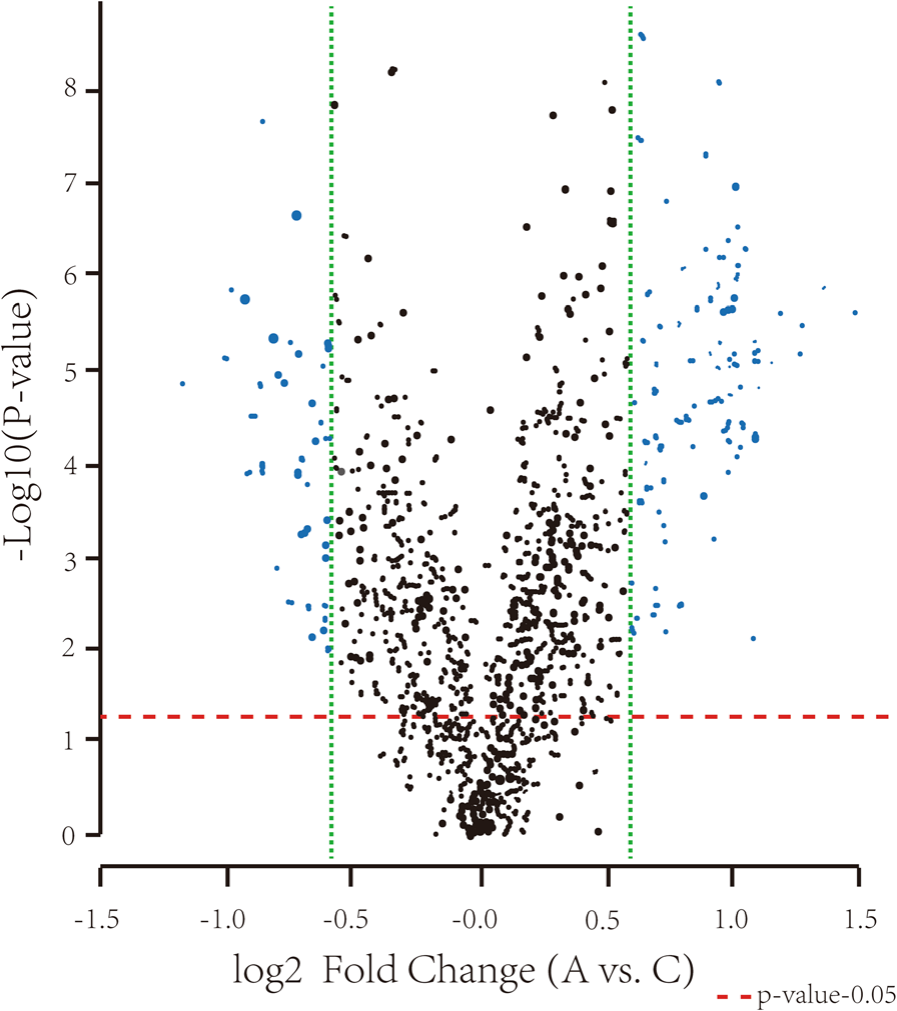
Volcano plot of miRNA expression differences between Aβ25–35 and control group. Blue spots represent genes which were differentially expressed

To investigate whether miRNAs involved in Aβ_25–35_ induced SH-SY5Y cell damage, differential expression of miRNAs was tested by HmiOA7.1 miRNA gene chip. Prior to the testing, purity and quality of samples need to be detected by Nanodrop ND-1000. The standard used is a ratio of A260/A280 equal or above 1.6, and A260/A230 equal or above 1. Meanwhile, integrality of samples should to be checked according to RIN equal o/Cllr above 5 (RIN > =5 means perfect integrality)by RNA6000 Nano RNA assay. The results indicated that every group has reached eligible standard (Fig. 2). We next identified differentially expressed genes in each contrast using an *P* value of below 0.05 and log2|Fold Change| equal or above 0.585 to capture the widest possible number of differentially expressed genes [11]. After profiling the expression of total 2588 mature miRNAs. 155 miRNAs were positively detected in our sample. For 15 miRNAs, we did find detectable negative traces (Fig. 3). Significant miRNAs (miR-210, miR-29, miR-197 and so on) have been reported as related to AD [12–14].

### Validation of the microarray data by quantitative PCR analysis

**Fig. 4:**
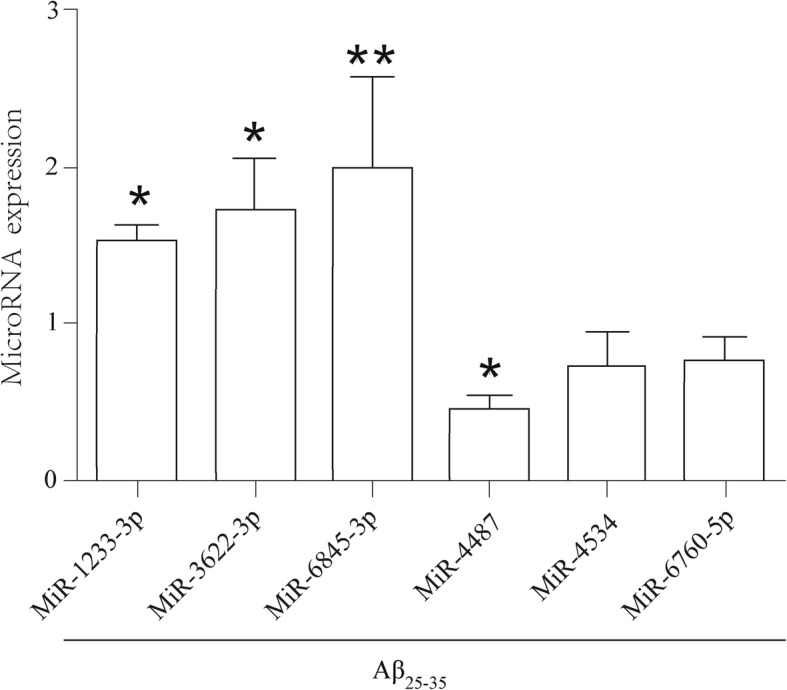
Aβ25–35 induced changes of miRNA level detected by qPCR *p < 0.05, **p < 0.01 vs. control; *n* = 3

To validate the miRNAs identified by microarray chip, according to abundance of miRNAs expression and functional significance, the previous-mentioned 6 candidate reference miRNAs (miR-6845-3p, miR-3622-3p, miR-1233-3p, miR-4487, miR-4534, and miR-6760-5p) and U6 were included in further confirmation phase by quantitative RT-PCR. As a result, these expression of dysregulation miRNAs were consistent with the microarray results (Fig. 4). Importantly, the level of miR-4487 was significantly down-regulated twice in the Aβ group compared to the control group, while expression of miR-6845-3p (*P* < 0.01) obviously up-regulated before miR-1233-3p (*P* < 0.05) and miR-3622-3p (*P* < 0.05). Lastly, based on both microarray and PCR studies, miR-6845-3p and miR-4487 appear as the most promising biomarkers involved in the process of Aβ cellular damage.

### Genes associated to the disease progression: Functional and biological findings

Gene Ontology (GO) enrichment and KEGG pathway analysis were adopted to discover the most remarkable functional significance miRNAs on DAVID websites by analyzing the target genes intersection of miRwalk. GO annotation analysis showed that candidate target genes of miR-4487 were distributed into apoptotic signaling pathway, intracellular receptor signaling peptide pathway, actin cytoskeleton and so on. The majority of the enriched KEGG terms were involved in the metabolic process. Emerging reports describe Aβ25–35 as possibly resulting in cell apoptosis. The up-regulated mir-6845-3p was also analyzed in GO analysis. Gene enrichment including development of central nervous, cell adhesion, guidance molecular of axon was determined in GO analysis. Certain KEGG pathways, for example the axon guidance molecules pathway, cell adhesion and cell motility were directly associated with mir-6845-3p, and they may affect the expression of mir-6845-3p. in total, these results were kept with the highest enriched GOs targeted and KEGG pathways by mir-6845-3p and mir-4487 miRNAs. Thus, our study will further verify that has-miR-4487 is involved in apoptosis resulted from Aβ25–35 and that has-miR-6845-3p participated in axonal regeneration.

### The miR-4487 reduced apoptosis in SH-SY5Y cell after Aβ25–35 treatment

**Fig. 5:**
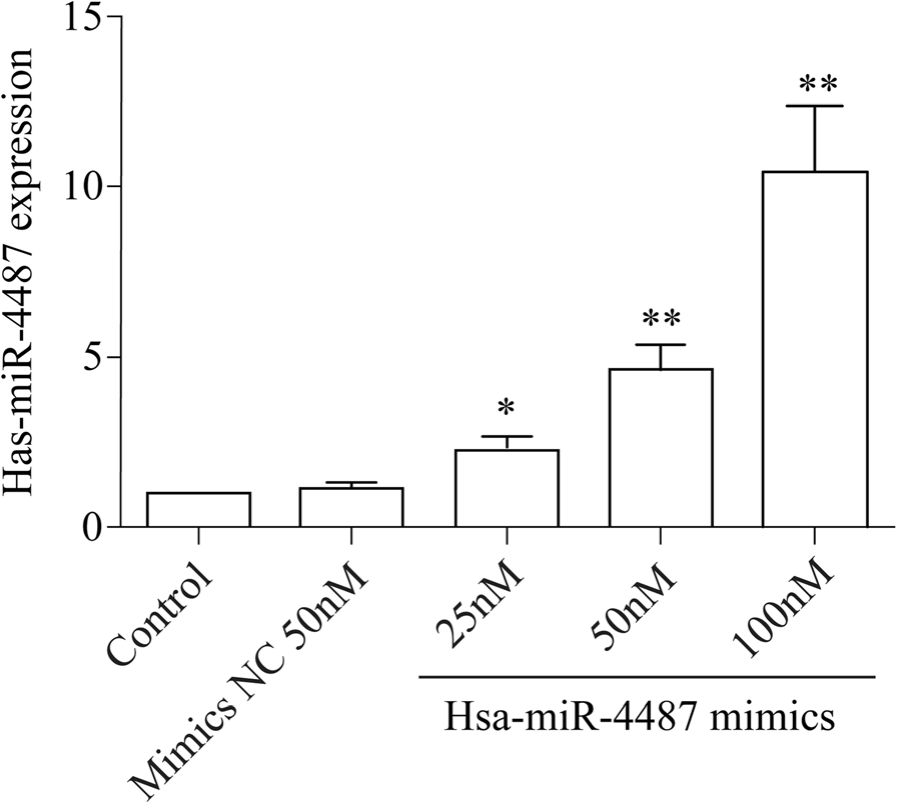
the effect on increase miRNA level of hsa-miR-4487 mimics detected by Real-time PCR *p < 0.05, **p < 0.01 vs. mimics NC

**Fig. 6:**
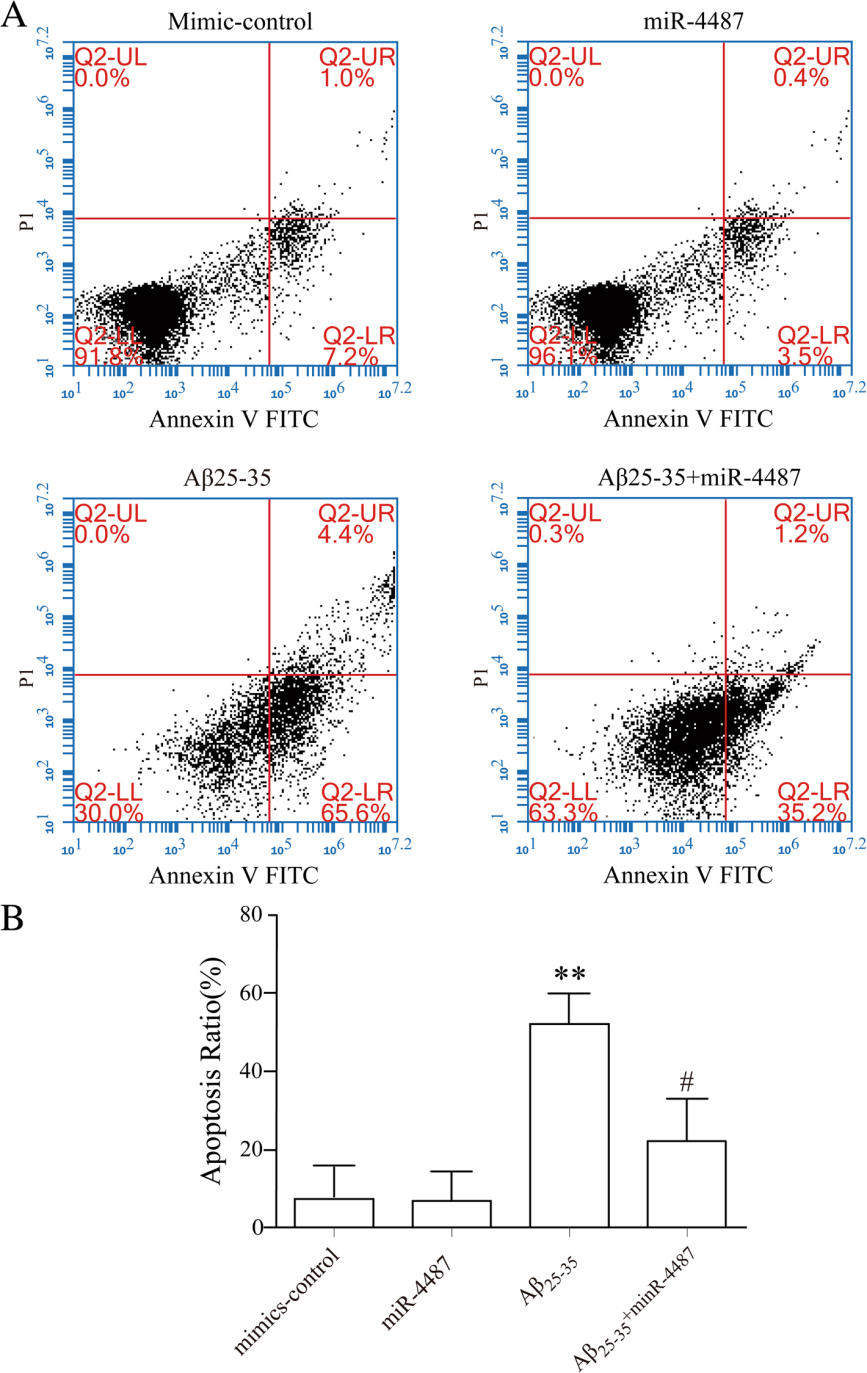
Hsa-miR-4487 mimics suppression SH-SY5Y cells apoptosis induced by Aβ25–35. **a** The representative pictures of apoptosis detected by Flow cytometry. **b** The summary data of apoptosis ratio (*N* = 3). *p < 0.05, **p < 0.01 vs. control; #*p* < 0.05 vs. Aβ25–35)

To verify the function of miR-4487 in cell apoptosis, concentrations of 25 nM, 50 nM, 100 nM SH-SY5Y cells were transfected with miR-4487 mimic as previously described respectively. Three days later, all cells stably overexpressed miR-4487, in which concentration of 50 nM, 100 nM SH-SY5Y cells were 4.62 and 10.45 times more than control (Fig. 5). The result showed that has-miR-4487 mimic possessed stably transfection efficiency. Following transfection, flow cytometry was conducted to examine levels of cell apoptosis affected by has-miR-4487. Analysis data indicated that the expression of apoptosis rate has no statistical significance compared has-miR-4487 mimics group with the mimics-control group. While the present study transfected miR-4487 mimics into SH-SY5Y cells.The apoptosis levels in SH-SY5Y cells significantly decreased from 54.74 to 21.11% (*P* < 0.05), as compared with no transfected miR-4487 mimics in SH-SY5Y cell lines treated with Aβ25–35 investigated (Fig. 6). These findings suggest that miR-4487 may serve a critical role in reducing the level of apoptosis levels in SH-SY5Y cells treated with Aβ25–35 who showed a strong cell impairment.

### The miR-6845-3p reduced cell axonal outgrowth involved in Aβ25–35 damaging model

**Fig. 7:**
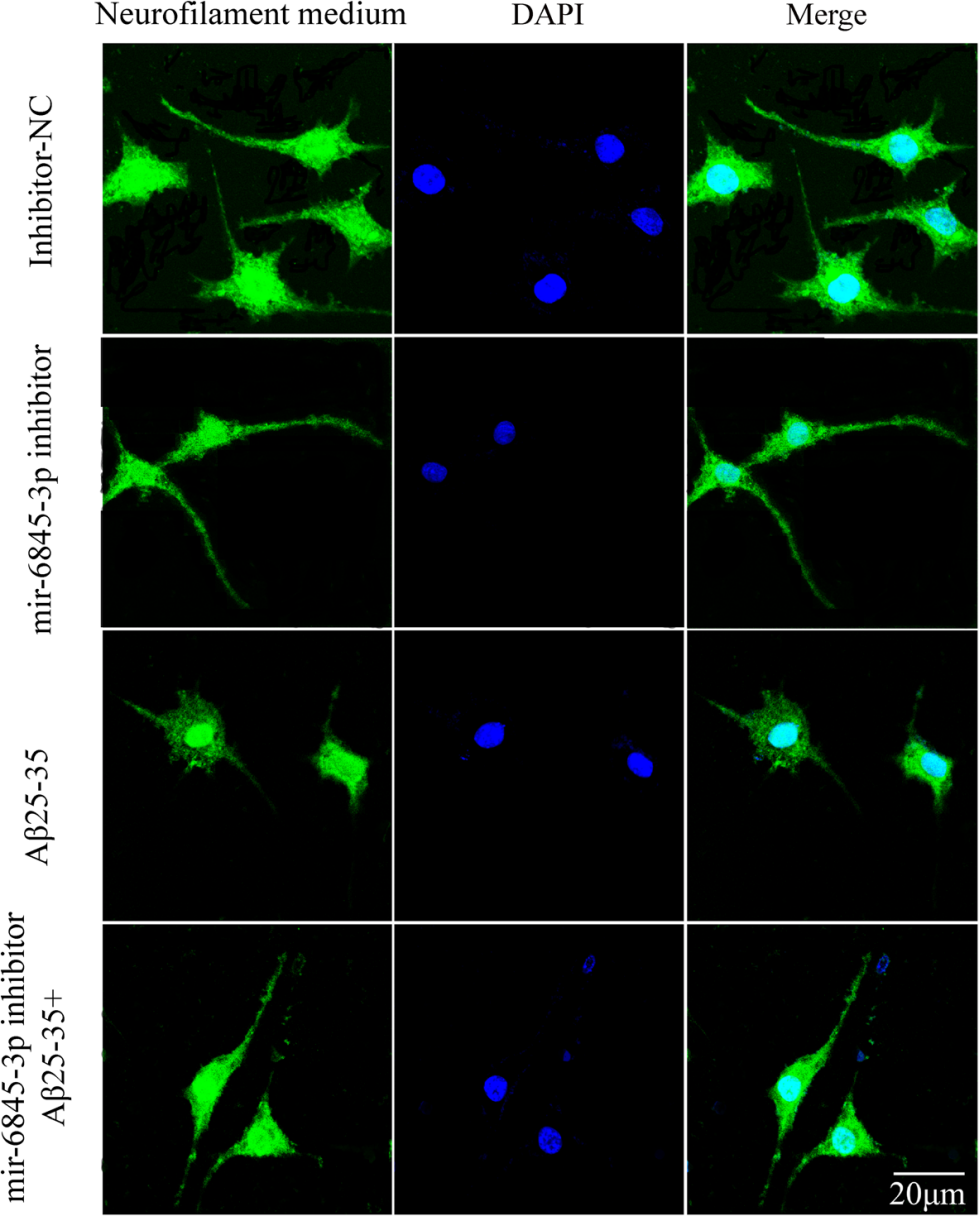
the effect of hsa-miR-6845-3p inhibitor on axon

Biological analysis predicted effects of hsa-miR-6845-3p focused on cell axon. Much of the research in cell impairment has examined that Aβ25–35 broke down cytoskeletons consisting of axon. Neurofilament, as the core of the axon, plays an important role in the formation of cell cytoskeletons [15]. Thus, based on this evidence, the expression of neurofilament protein and cellular morphology need to be measured to detect the role of hsa-miR-6845-3p in Aβ25–35 damaging model by confocal laser scanning microscopy. When hsa-miR-6845-3p inhibitor was transfected into SH-SY5Ycell, the average neuronal neurite length was found to extend in contrast with control group (inhibitor- NC). As shown in Fig. 7, Aβ_25–35_ can be significantly induced, not only limited to the cell body, but also present on the neuron extension. Moreover, in order to precisely verify the function of hsa-miR-6845-3p on these neurons, we transfected the hsa-miR-6845-3p inhibitor into SH-SY5Y cell treated with Aβ_**25–35.**_ Interestingly, we observed longer and greater effect on cell axon in hsa-miR-6845-3p inhibitor added to Aβ_25–35_ group (Fig. 7). This result coincided with previous primary consequence, which have confirmed increased expression of hsa-miR-6845-3p in SH-SY5Y cell treated with Aβ_**25–35.**_ Taken together, these findings support the view that higher expression of hsa-miR-6845-3p are likely to inhibit neurite extension and vice versa if cells were in the situation of decreased expression of hsa-miR-6845-3p, axon length might be improved in SH-SY5Y cell. Thus, we hypothesize that Aβ_**25–35**_ might damage cytoskeletons via function of hsa-miR-6845-3p.

## Discussion

As the principal cognitive disorder problem in humans, the incidence rate of AD increased significantly with the aging population [16]. Since there are neither effective therapeutic strategies nor early diagnostic biomarkers for the treatment of AD [17], finding novel strategies of early diagnosis and treatment for AD has long been a central goal. miRNAs allow novel insight into modification mechanisms employed from expression ability of miRNAs to function study in AD and can be easily detected by microarray analysis or RT-PCR. Therefore, miRNAs could potentially serve as noninvasive biomarkers and therapeutic targets of AD [18–20]. Besides, our research also reveals gaps in knowledge that require further research.

Neurofibrillary tangles are one of the vital pathological characteristics of AD [21], and the accumulation of Aβ_**25–35**_ can cause and exacerbate the production of neurofibrillary tangles [22–24]. In our study, we focused primary on whether promising miRNAs could act as accurate biomarkers to discriminate AD from normal cases by taking advantage of miRNA array data sets. We proved that SH-SY5Y cell in combination with treated with concentration 40um/L Aβ_**25–35**_ was efficiency of cell impaired models of AD, several of report has also proved efficiency used to establish of this model [25]. Meanwhile, our studies analyzed gene chip and Qpcr experiment rely on this model to identify two significant miRNAs, down-regulation miR-4487 and up-regulation miR-6845-3P, involved in the impairment of SH-SY5Y cell treated with Aβ; furthermore, Gene Ontology (GO) enrichment and KEGG pathway analysis were adopted to discover the most remarkable, functionally significant miRNAs.Here, based on the results of these analysis, we found that the functions of most differentially regulated genes of miR-4487 and miR-6845-3p were related to the regulation of cellular component organization, protein import into nucleus, glucose metabolic process, and so forth, in which apoptotic *P*-value of signaling pathway involved in miR-4487 is higher significance, indicating that miR-4487 might participate in the dysfunction of apoptotic signaling pathway. In addition, emerging research showed that up-regulated miR-4487 can promote cell apoptosis and proliferation in salivary adenoid cystic carcinoma and early-stage colorectal cancer [26, 27]. Dysregulation miR-4487 can modulating autophagy in PD [28].

Neuronal apoptosis is a key pathological feature of AD [29]. In the early stage of AD, accumulated Aβ protein will result in a loss of microbial anabolism and catabolism, and cell apoptosis response worsens with the progression of the disease itself [30]. To investigate the influence of miR-4487 in cell apoptosis, we transfected miR-4487 mimic into SH-SY5Y cell, which could strengthen regulation of miRNA. We found that miR-4487 increased expression relieves the level of apoptosis in Aβ25–35 treated SH-SY5Y neuronal cells. in addition, our study provides the first evidence that miR-4487 may be an effective strategy to modulate cell apoptosis levels attributed to Aβ reduction. Similarly, bioinformatic analysis revealed the regulation of miR-6845-3p associated with neuronal maintenance and synaptic plasticity. However, no reports have been published that linked miR-6845-3p to AD, only that miR-6845-3p was located in chromosome 8q24.3. meaningAβ (Amyloid-β) can hold back nerve reconstruction and neuron extension in brains of Alzheimer’s patients [31]. Given prior evidence supporting the idea that the miR-6845-3p gene could influence axonal outgrowth, we speculate that miR-6845-3p might participate in axon elongation disorder arising from Aβ25–35. In view of this, a further experiment, constructing of miR-6845-3p inhibitor into SH-SY5Y cell to observe axonal variations, is conducted to detect expression of neurofilament, down-expression of proteins maintaining cell cytoskeleton stability, and response to axonal conditions; our results took the lead in suggesting that decreased miR-6845-3p expression promotes significant axonal outgrowth and vice versa. These effects might be associated, in part, to a direct regulation of Aβ25–35 at the miR-6845-3p level.

Overall, the two selected miRNAs as potential diagnostic panel for AD are among the top ones of 6 investigated miRNAs which were functionally mapped to proteins involved in AD pathology by our two different bioinformatics searches in the databases of miRwalk and DAVID. Our study serves as a proof-of-concept that findings significant miR-4487 and miR-6845-3p may participate in AD occurrence and development by effective strategy to modulate cell apoptosis levels and axon elongation. in addition, our study provides the first evidence that miR-6845-3p may be an effective strategy to modulate cell axon elongation attributed to Aβ reduction. Furthermore, we used online software containing predictive (TargetScan) and experimentally validated (MiRTarBase) miRNA targets to find that ULK1 may be a potential common target gene of miR-4487 and miR-6845-3p, which could negatively or positively regulate ULK1. A putative miR-4487 binding site exists in the 3’-UTR of ULK1 mRNA and seven point mutations were generated in the binding site. Recently, in reported study, we found that ULK1 activation could inhibit the expression of p-p70^S6K^ or p-eEF2K. We also found that si-p70^S6K^ had no effect on ULK1 but si-ULK1could decrease p-p70^S6K^ expression [32, 33], suggesting that ULK1 may negatively regulate p70^S6K^ in SH-SY5Y cell autophagy. Autophagy plays an important role in nervous system disease [34], and other emerging studies have reported that autophagy, as a conserved homeostatic process for degrading long-lived proteins and damage organelles, may contribute to neurodegeneration in PD [28]. In addition, silencing of eEF2K has been reported to promote autophagic survival via indirect activation of the AMPK-ULK1 pathway in colon cancer cells [35]. However, our results may uncover the novel ULK1 autophagic pathway, as well as a correlation of the levels of the miR-4487 and miR-6845-3p, analyzing both with the new ULK1 target mRNA. This necessitates further studies to gain insight in the detail mechanism of the effects of the two miRNAs target genes in SH-SY5Y cells and into the function of these two miRNAs in AD rats. Although, we tried to avoid bias in our study, other certain limitations still need to be considered while interpreting the result of our study. First, a microarray data set of miRNAs in the included cell studies was selected. More useful miRNAs should be obtained to verify accuracy of previous study by downloading a set of raw microarray data. Second, to confirm the variation in two miRNAs expression at different stage of AD, different stages (1-month-old, 3-month-old, 6-month-old, and 9-month-old) of AD rats should be used for experimental validation. This tissue-based study will lay the foundation of further development of blood-based diagnostics/therapeutic novel biomarker candidates of AD. Moreover, as mentioned above, an analysis of the putative functional targets of miRNAs and their target gene experimental validation can provide input in deciphering AD pathogenesis. Bridging the gap between significant miRNAs and autophagy would be impressive for better understanding of their intricate relationships and thus providing a novel diagnostics/therapeutic strategy in AD. Thus, these findings would provide a clue to explore significantly miRNAs and its target ULK1as potential biomarkers in the future AD diagnosis/therapy.

## Conclusions

Our study not only demonstrate that miR-4487and miR-6845-3p may participate in AD occurrence and development and provide the first evidence that miR-6845-3p may be an effective strategy to modulate cell axon elongation attributed to Aβ reduced, but it also contributes to greater insight on new potential mechanisms and functions for predicting AD. Together, further in-depth miRNA research and discovery will provide novel promising ideas and strategies for elucidating the pathological mechanisms of AD and for developing effective methods for early diagnosis and treatment of AD.

## Abbreviations

AD: Alzheimer’s disease.
ATCC: American type culture collection
Aβ: Accumulation of amyloid β-peptide
DMEM: glucose modified Eagle’s medium,
GO: Gene Ontology
KEGG: Kyoto Encyclopedia Genes and Genomes
miRNAs: microRNAs,
qRT-PCR: Quantitative real-time Reverse Transcriptase Polymerase Chain Reaction

## References

[1] Abe M, Bonini NM (2013). MicroRNAs and neurodegeneration: role and impact. Trends Cell Biol.

[2] Rivera I, Capone R, Cauvi DM, Arispe N, De Maio A (2017). Modulation of Alzheimer's amyloid beta peptide oligomerization and toxicity by extracellular Hsp70. Cell Stress Chaperones.

[3] Wei CW, Luo T, Zou SS, Wu AS (2017). Research progress on the roles of microRNAs in governing synaptic plasticity, learning and memory. Life Sci.

[4] Karthikeyan A, Patnala R, Jadhav SP, Eng-Ang L, Dheen ST (2016). MicroRNAs: key players in microglia and astrocyte mediated inflammation in CNS pathologies. Curr Med Chem.

[5] Zhao J, Yue D, Zhou Y, Jia L, Wang H, Guo M, Xu H, Chen C, Zhang J, Xu L (2017). The role of MicroRNAs in Abeta deposition and tau phosphorylation in Alzheimer's disease. Front Neurol.

[6] Song J, Kim YK (2017). Identification of the role of miR-142-5p in Alzheimer's disease by comparative bioinformatics and cellular analysis. Front Mol Neurosci.

[7] Reddy PH, Tonk S, Kumar S, Vijayan M, Kandimalla R, Kuruva CS, Reddy AP (2017). A critical evaluation of neuroprotective and neurodegenerative MicroRNAs in Alzheimer's disease. Biochem Biophys Res Commun.

[8] Blonz ER. Alzheimer's disease as the product of a progressive energy deficiency syndrome in the central nervous system: the Neuroenergetic hypothesis. J Alzheimers Dis. 2017. 10.3233/JAD-170549 PMC5676979 28946565

[9] Jha SK, Jha NK, Kumar D, Sharma R, Shrivastava A, Ambasta RK, Kumar P (2017). Stress-induced synaptic dysfunction and neurotransmitter release in Alzheimer's disease: can neurotransmitters and neuromodulators be potential therapeutic targets?. J Alzheimers Dis.

[10] Zhang Y, Pan HY, Hu XM, Cao XL, Wang J, Min ZL, Xu SQ, Xiao W, Yuan Q, Li N (2016). The role of myocardin-related transcription factor-a in Abeta25-35 induced neuron apoptosis and synapse injury. Brain Res.

[11] Hu L, Ai J, Long H, Liu W, Wang X, Zuo Y, Li Y, Wu Q, Deng Y (2016). Integrative microRNA and gene profiling data analysis reveals novel biomarkers and mechanisms for lung cancer. Oncotarget.

[12] Ma Q, Dasgupta C, Li Y, Bajwa NM, Xiong F, Harding B, Hartman R, Zhang L (2016). Inhibition of microRNA-210 provides neuroprotection in hypoxic-ischemic brain injury in neonatal rats. Neurobiol Dis.

[13] Pereira PA, Tomas JF, Queiroz JA, Figueiras AR, Sousa F (2016). Recombinant pre-miR-29b for Alzheimer s disease therapeutics. Sci Rep.

[14] Willeit P, Zampetaki A, Dudek K, Kaudewitz D, King A, Kirkby NS, Crosby-Nwaobi R, Prokopi M, Drozdov I, Langley SR (2013). Circulating microRNAs as novel biomarkers for platelet activation. Circ Res.

[15] Fialova L, Bartos A, Svarcova J (2017). Neurofilaments and tau proteins in cerebrospinal fluid and serum in dementias and neuroinflammation. Biomed Pap Med Fac Univ Palacky Olomouc Czech Repub.

[16] Bhardwaj D, Mitra C, Narasimhulu CA, Riad A, Doomra M, Parthasarathy S (2017). Alzheimer's disease-current status and future directions. J Med Food.

[17] Willen K, Sroka A, Takahashi RH, Gouras GK (2017). Heterogeneous Association of Alzheimer's disease-linked Amyloid-beta and Amyloid-beta protein precursor with synapses. J Alzheimers Dis.

[18] Reddy PH, Williams J, Smith F, Bhatti JS, Kumar S, Vijayan M, Kandimalla R, Kuruva CS, Wang R, Manczak M (2017). MicroRNAs, aging, cellular senescence, and Alzheimer's disease. Prog Mol Biol Transl Sci.

[19] Prasad KN (2017). Oxidative stress and pro-inflammatory cytokines may act as one of the signals for regulating microRNAs expression in Alzheimer's disease. Mech Ageing Dev.

[20] Lusardi TA, Phillips JI, Wiedrick JT, Harrington CA, Lind B, Lapidus JA, Quinn JF, Saugstad JA (2017). MicroRNAs in human cerebrospinal fluid as biomarkers for Alzheimer's disease. J Alzheimers Dis.

[21] Metaxas A, Kempf SJ (2016). Neurofibrillary tangles in Alzheimer's disease: elucidation of the molecular mechanism by immunohistochemistry and tau protein phospho-proteomics. Neural Regen Res.

[22] Gilbert J, Shu S, Yang X, Lu Y, Zhu LQ, Man HY (2016). beta-amyloid triggers aberrant over-scaling of homeostatic synaptic plasticity. Acta Neuropathol Commun.

[23] Barone FC, Gustafson D, Crystal HA, Moreno H, Adamski MG, Arai K, Baird AE, Balucani C, Brickman AM, Cechetto D (2016). First translational 'Think Tank' on cerebrovascular disease, cognitive impairment and dementia. J Transl Med..

[24] Thaker AA, Weinberg BD, Dillon WP, Hess CP, Cabral HJ, Fleischman DA, Leurgans SE, Bennett DA, Hyman BT, Albert MS (2017). Entorhinal cortex: Antemortem cortical thickness and postmortem neurofibrillary tangles and amyloid pathology. AJNR Am J Neuroradiol.

[25] Lee S, Youn K, Jeong WS, Ho CT, Jun M (2017). Protective effects of red ginseng oil against Abeta25-35-induced neuronal apoptosis and inflammation in PC12 cells. Int J Mol Sci.

[26] Chen W, Zhao X, Dong Z, Cao G, Zhang S (2014). Identification of microRNA profiles in salivary adenoid cystic carcinoma cells during metastatic progression. Oncol Lett.

[27] Ghanbari R, Mosakhani N, Asadi J, Nouraee N, Mowla SJ, Poustchi H, Malekzadeh R, Knuutila S (2015). Decreased expression of fecal miR-4478 and miR-1295b-3p in early-stage colorectal cancer. Cancer Biomark.

[28] Chen Y, Wang S, Zhang L, Xie T, Song S, Huang J, Zhang Y, Ouyang L, Liu B (2015). Identification of ULK1 as a novel biomarker involved in miR-4487 and miR-595 regulation in neuroblastoma SH-SY5Y cell autophagy. Sci Rep.

[29] Ramos-Martinez I, Martinez-Loustalot P, Lozano L, Issad T, Limon D, Diaz A, Perez-Torres A, Guevara J, Zenteno E (2017). Neuroinflammation induced by amyloid beta25-35 modifies mucin-type O-glycosylation in the rat's hippocampus. Neuropeptides.

[30] Huang SW, Wang W, Zhang MY, Liu QB, Luo SY, Peng Y, Sun B, Wu DL, Song SJ (2016). The effect of ethyl acetate extract from persimmon leaves on Alzheimer's disease and its underlying mechanism. Phytomedicine.

[31] Colin J, Allouche A, Chauveau F, Corbier C, Pauron-Gregory L, Lanhers MC, Claudepierre T, Yen FT, Oster T, Malaplate-Armand C (2016). Improved neuroprotection provided by drug combination in neurons exposed to cell-derived soluble amyloid-beta peptide. J Alzheimers Dis.

[32] Wang B, Kundu M (2017). Canonical and noncanonical functions of ULK/Atg1. Curr Opin Cell Biol.

[33] Jang W, Kim HJ, Li H, Jo KD, Lee MK, Yang HO (2016). The neuroprotective effect of erythropoietin on rotenone-induced neurotoxicity in SH-SY5Y cells through the induction of autophagy. Mol Neurobiol.

[34] Wnuk A, Kajta M (2017). Steroid and xenobiotic receptor Signalling in apoptosis and autophagy of the nervous system. Int J Mol Sci.

[35] Xie CM, Liu XY, Sham KW, Lai JM, Cheng CH (2014). Silencing of EEF2K (eukaryotic elongation factor-2 kinase) reveals AMPK-ULK1-dependent autophagy in colon cancer cells. Autophagy.

